# CHIP regulates bone mass by targeting multiple TRAF family members in bone marrow stromal cells

**DOI:** 10.1038/s41413-018-0010-2

**Published:** 2018-03-29

**Authors:** Tingyu Wang, Shan Li, Dan Yi, Guang-Qian Zhou, Zhijie Chang, Peter X. Ma, Guozhi Xiao, Di Chen

**Affiliations:** 1 Department of Pharmacy, Shanghai Ninth People’s Hospital, Shanghai JiaoTong University School of Medicine, 200011 Shanghai, China; 2 Department of Orthopedic Surgery, Rush University Medical Center, Chicago, IL 60612 USA; 3 Department of Medical Cell Biology and Genetics, Shenzhen Key Laboratory and the Center for Anti-Ageing and Regenerative Medicine, Shenzhen University Medical School, 518060 Shenzhen, China; 4 State Key Laboratory of Biomembrane and Membrane Biotechnology, Tsinghua University School of Medicine, 100084 Beijing, China; 5 Department of Biologic and Materials Science, University of Michigan, Ann Arbor, MI 48109 USA

## Abstract

Carboxyl terminus of Hsp70-interacting protein (CHIP or STUB1) is an E3 ligase and regulates the stability of several proteins which are involved in different cellular functions. Our previous studies demonstrated that *Chip* deficient mice display bone loss phenotype due to increased osteoclast formation through enhancing TRAF6 activity in osteoclasts. In this study we provide novel evidence about the function of CHIP. We found that osteoblast differentiation and bone formation were also decreased in *Chip* KO mice. In bone marrow stromal (BMS) cells derived from *Chip*^−/−^ mice, expression of a panel of osteoblast marker genes was significantly decreased. ALP activity and mineralized bone matrix formation were also reduced in *Chip-*deficient BMS cells. We also found that in addition to the regulation of TRAF6, CHIP also inhibits TNFα-induced NF-κB signaling through promoting TRAF2 and TRAF5 degradation. Specific deletion of *Chip* in BMS cells downregulated expression of osteoblast marker genes which could be reversed by the addition of NF-κB inhibitor. These results demonstrate that the osteopenic phenotype observed in *Chip*^−/−^ mice was due to the combination of increased osteoclast formation and decreased osteoblast differentiation. Taken together, our findings indicate a significant role of CHIP in bone remodeling.

## Bone remodeling: protein promise for bone loss disorders

A protein involved in maintaining balance in bone formation may prove a useful target for treating bone loss-associated diseases. Bones continuously undergo formation and resorption, but certain diseases can interfere with this remodeling process, leading to loss of bone mass. Previous studies by Di Chen at Rush University Medical Center in Chicago, US, and co-workers found that mice without a key protein called CHIP display increased osteoclast formation and abnormally high levels of bone resorption. Further investigations by Chen’s team now indicate that CHIP deficiency also results in reduced bone formation and loss of mass. They found that CHIP regulates a family of proteins called TRAF—the overexpression of which disturbs the precise balance of osteoclast/osteoblast bone cell formation. CHIP may provide a target for drug development for bone loss diseases.

## Introduction

Bone remodeling is a dynamic process, involving a balance between the activity of bone-forming osteoblasts, derived from mesenchymal progenitor cells, and the activity of bone-resorbing osteoclasts, derived from hematopoietic stem cells.^1^ A disruption of this balance leads to many bone and joint diseases, especially metabolic bone diseases. Although the coupling of bone resorption and bone formation has long been recognized, the mechanism mediating this fundamental process in skeletal homeostasis is not fully understood. The understanding of the regulatory mechanisms of osteoclast formation and osteoblast function is critical for the rational drug design for the treatment of metabolic bone disorders.

Osteoclasts are derived from hematopoietic stem cells which undergo proliferation and differentiation to become multinucleated bone-resorbing osteoclasts.^2^ The process of osteoclastogenesis is regulated by several cytokines such as RANKL and TNF-α,^3^ which activate NF-κB signaling.^2^ TNF-α receptor-associated factors (TRAFs) are intracellular adapter proteins, which play critical roles in NF-κB signaling.^4,5^ In TRAF family members, TRAF2, TRAF5, and TRAF6 have been shown to activate NF-κB signaling.^6^ TRAF2 and TRAF5 interact with TNF-α receptors and mediate TNF-α-induced osteoclast formation. In *Traf2* deficient cells, TNF-α-induced osteoclast formation was severely impaired.^7^ Similar results were also found in *Traf5*-deficient bone marrow stromal (BMS) cells.^8^ Therefore, both TRAF2 and TRAF5 are essential for TNF-α-induced osteoclast formation.^7,8^ Moreover, TRAF6 interacts with RANK to activate NF-κB signaling which in turn regulates osteoclast formation.^2,9–11^ Multiple TRAF family members, including TRAFs 2, 3, 5, and 6, have been demonstrated to play an important role in regulation of NF-κB signaling.^4–11^ In previous studies, we examined the effect of CHIP on TRAF6 protein stability.^12^ In the present studies we further examined the effects of CHIP on protein stability of TRAFs 2, 3, and 5. We found that in addition to TRAF6, CHIP also induced the degradation of TRAF2 and TRAF5, but not TRAF3.

Osteoblast is a cell type derived from mesenchymal progenitor cells and is responsible for bone formation. Osteoclast and osteoblast are two major cell types involved in bone remodeling in trabecular bone. Several previous studies have demonstrated that NF-κB signaling plays critical roles in regulation of osteoclast and osteoblast activities in bone. Activation of NF-κB signaling promotes osteoclast formation^13–16^ and inhibits osteoblast function.^17–22^

CHIP is an E3 ligase, which has been reported to promote ubiquitination and degradation of several substrates involving in different signaling pathways.^23–25^ In vivo findings demonstrated that *Chip* deficiency causes increased sensitivity to stress associated hyperthermia,^26^ leading to a markedly reduced life span in mice.^27^ In addition, *Chip*^−/−^ mice developed severe defects in heart and neural tissues.^28,29^ Moreover, *Chip*^−/−^ mice exhibit defects in motor sensory, cognitive, and reproductive function.^30^ Although the mechanisms of NF-κB regulation during bone remodeling have been extensively investigated, the roles of CHIP in regulation of NF-κB signaling and osteoblast function has not been demonstrated. Our previous study demonstrated that *Chip*^−/−^ mice develop osteopenic phenotype due to increased osteoclast formation.^12^ In the present studies, we investigated the roles of CHIP in regulation of protein stability of multiple TRAF family members, NF-κB signaling, and osteoblast function. Our findings provide new insights into the role of CHIP in regulation of osteoclast and osteoblast functions.

## Results

### Bone mass is decreased in *Chip*^−/−^ mice

**Fig. 1:**
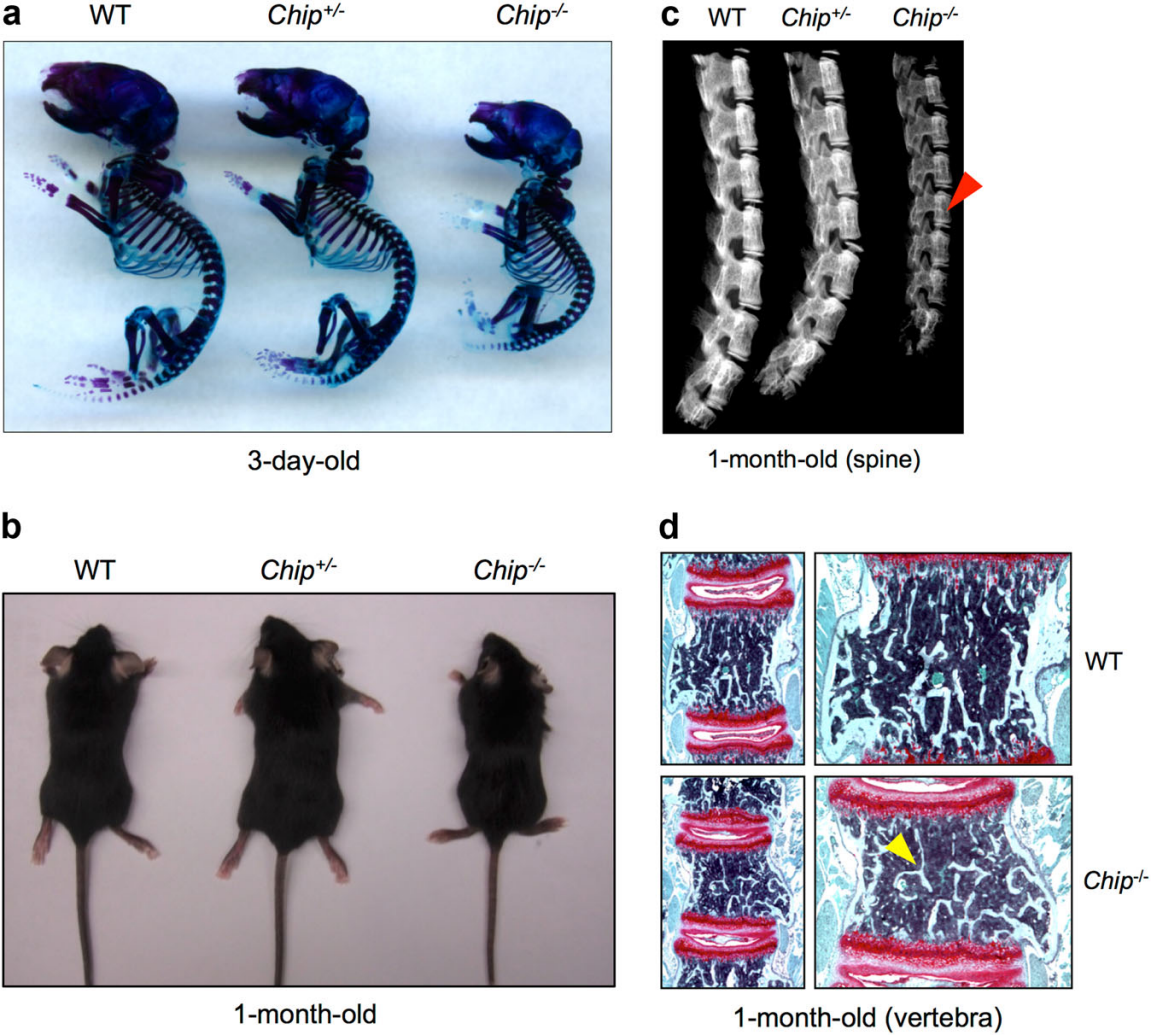
Bone mass is decreased in *Chip*KO mice. **a** The skeleton of 3-day-old newborn mice was stained with Alizarin red and Alcian blue. The size of *Chip*^−/−^ mice was significantly smaller than those of WT and *Chip*^+/-^ mice. **b** The picture showed the appearance of 1-month-old *Chip* KO mice (*Chip*^−/−^ and *Chip*^+/-^) and WT littermate. The size of *Chip*^−/−^ mice was smaller than that of WT and *Chip*^+/-^ mice. **c** X-ray radiographic analysis showed that bone density of the spine of 1-month-old *Chip*^−/−^ mice was lower compared to that of WT and *Chip*^+/-^ mice (indicated by red arrowhead). **d** Representative images of Safranin O/fast green stained sections of vertebrae of 1-month-old *Chip*^−/−^ mouse and its WT littermate showing reduced bone mass in vertebral bone of *Chip*^−/−^ mice (indicated by yellow arrowhead).

To determine the contribution of CHIP in skeletal development, we performed whole skeletal Alizarin red/Alcian blue staining using postnatal 3-day-old wild-type (WT) and *Chip*KO mice. The sizes of the skeleton of *Chip*^−/−^ mice were much smaller than those of WT littermates and *Chip*heterozygous KO mice (*Chip*^+/-^) (Fig. 1a). We also analyzed the appearance of 1-month-old *Chip*^−/−^ mice. The sizes of *Chip*^−/−^ mice were also smaller than WT and *Chip*^+/-^ KO mice (Fig. 1b). The X-ray radiographic analysis showed that the length of the spine was shorter and bone density of vertebral bone was reduced in 1-month-old *Chip*^−/−^ mice compared to WT and *Chip*^+/-^ KO mice (Fig. 1c). To further confirm changes in the spine of *Chip*^−/−^ mice, Safranin O/Fast green staining was performed on vertebral sections. The results showed that vertebral bone volume in spine was significantly reduced in 1-month-old *Chip*^−/−^ mice compared to WT littermates (Fig. 1d). These results indicate that *Chip* deficiency in mice results in a bone loss phenotype.

### Alterations of osteoclast formation and osteoblast function in *Chip*^−/−^ mice

**Fig. 2:**
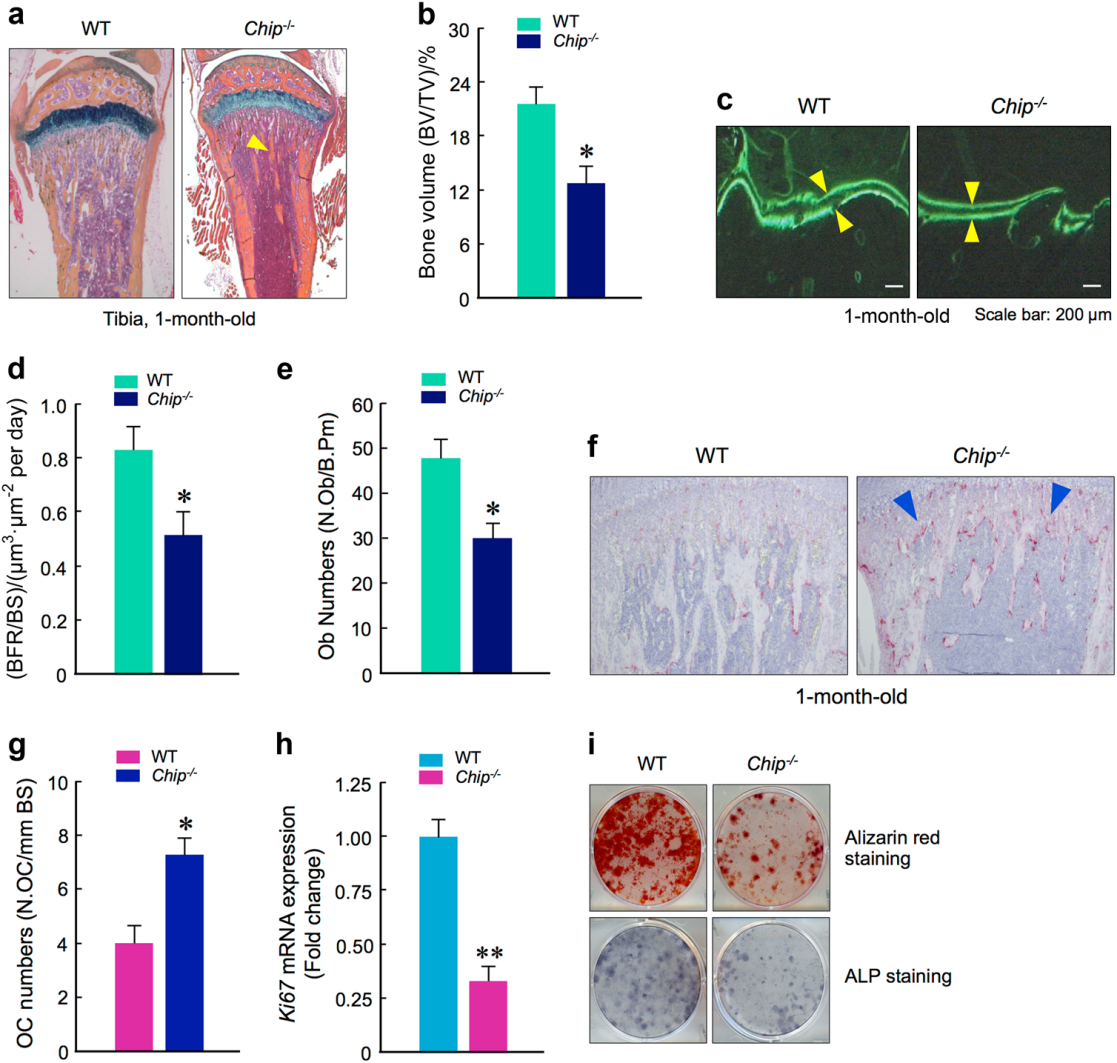
Reduction in bone formation and osteoblast function in *Chip*^−/−^ mice. **a, b** Representative images of Alcian blue/H&E Orange G stained tibiae sections from 1-month-old WT and *Chip*^−/−^ mice showed reduced bone volume in long bone (indicated by yellow arrowhead). **b** The quantification of bone volume was performed. **c**, **d** Calcein double labeling of trabecular bones in WT and *Chip*^−/−^ mice that were injected with calcein 4 and 1 day before the mice were sacrificed. Pictures of trabecular bone in tibiae were taken under the fluorescence microscope. *Chip*^−/−^ mice showed a decrease in bone formation rates (**d**). **e** Results of histomorphometric analysis also showed that osteoblast numbers were also significantly reduced in *Chip*^−/−^ mice. **f**, **g** TRAP staining was performed using tibial sections of 1-month-old WT and *Chip*^−/−^ mice. TRAP-positive cells were indicated by blue arrowheads. The numbers of TRAP-positive osteoclasts were higher in *Chip*^−/−^ mice (**g**). **h**, **i** Bone marrow stromal (BMS) cells were isolated from 1-month-old WT and *Chip*^−/−^ mice, and were cultured with ascorbic acid and β-glycerophosphate for 3 days for *Ki67* expression **(h**) and ALP staining (**i**). The BMS cells were also cultured with osteoblast differentiation medium in the presence of BMP-2 (50 ng·mL^-1^) for 2 weeks and Alizarin red staining was performed (**i**). *Ki67* expression, ALP activity, and mineralized bone matrix formation were significant decreased in *Chip*^−/−^ mice. *P < 0.05, **P < 0.01, unpaired Student’s *t*-test, *n* = 6.

The osteopenic phenotype observed in *Chip*^−/−^ mice could be due to either increased bone resorption or reduced bone formation. To determine the effect of *Chip* deficiency on bone formation, bone histology and the calcein double labeling assays were performed. The results showed that the bone volume and bone formation rates were significantly reduced in *Chip*^−/−^ mice (Fig. 2a-d). In addition, we also found that osteoblast numbers were significantly reduced in *Chip*^−/−^ mice (Fig. 2e). To determine the role of CHIP in bone resorption, we performed TRAP staining using histological sections of tibiae from 1-month-old WT and *Chip*^−/−^ mice. The data revealed that TRAP positive osteoclast numbers were significantly increased in *Chip*^−/−^ mice (Fig. 2f, g). These results suggest that the bone loss phenotype observed in *Chip*^−/−^ mice could be due to the combination of increased bone resorption and decreased bone formation. To further determine changes in osteoblast differentiation, we isolated BMS cells from 1-month-old WT and *Chip*^−/−^ mice, and examined *Ki67* expression by real-time PCR and performed alkaline phosphatase (ALP) and Alizarin red staining assays. The results showed that *Ki67* expression was significantly reduced and ALP activity and mineralized bone matrix formation were dramatically decreased in *Chip*-deficient BMS cells compared to BMS cells isolated from WT mice (Fig. 2h, i). These results further demonstrated that osteoblast function was reduced in *Chip*^−/−^ mice.

### CHIP regulates protein stability of multiple TRAF family members

**Fig. 3:**
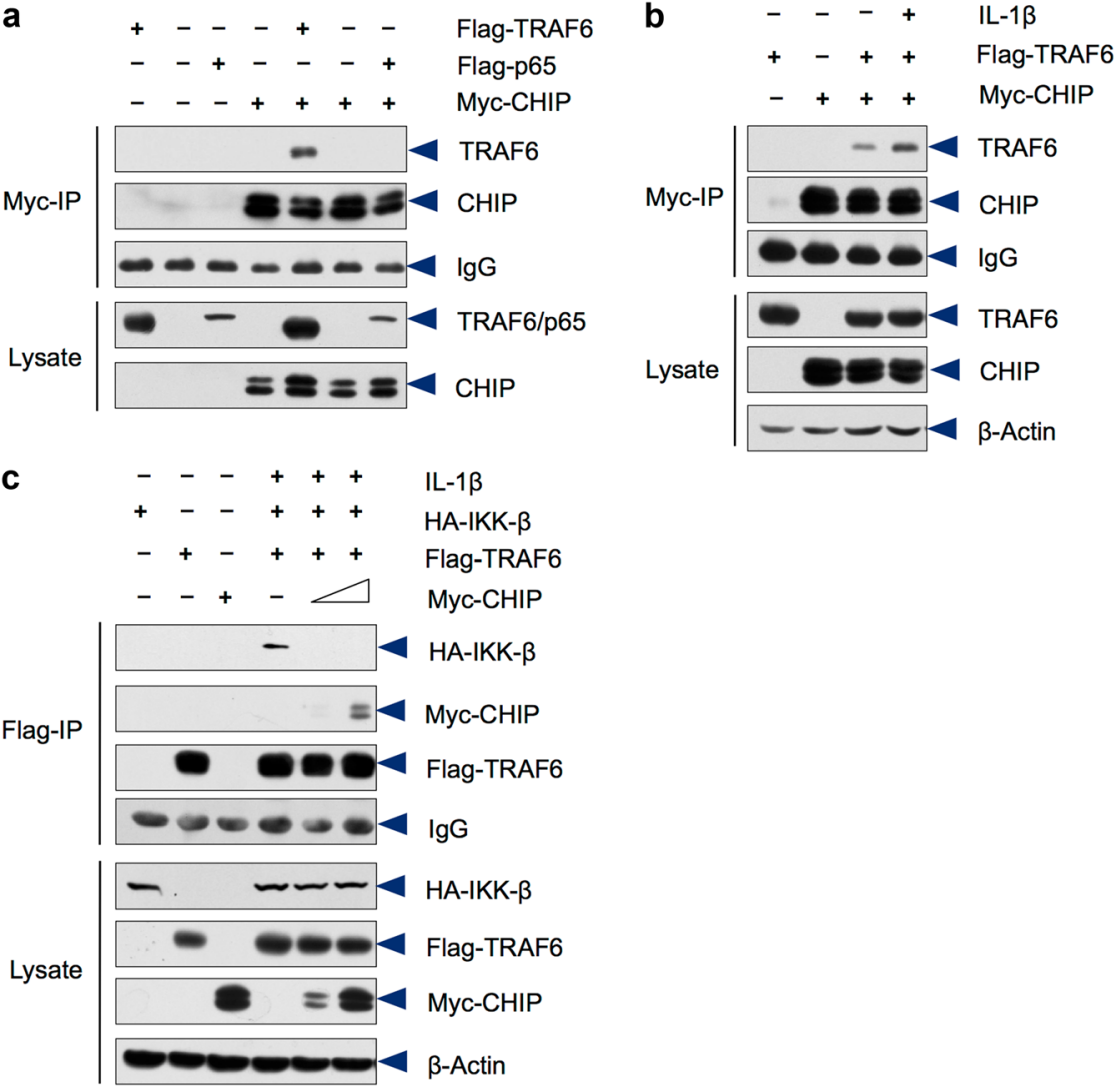
CHIP attenuates IL-1β-induced TRAF6/IKK-β complex formation. **a** Flag-TRAF6 and Flag-p65 were transfected into 293 T cells with or without Myc-CHIP. The immunoprecipitates were prepared with the anti-Myc antibody followed by the Western blot analysis. The data showed that CHIP interacts with TRAF6, but not p65. **b** IL-1β enhances CHIP/TRAF6 interaction. 293 T cells were transfected with the Flag-TRAF6 and Myc-CHIP as indicated and treated with IL-1β. The cells lysates were immunoprecipitated with the anti-Myc antibody followed by the Western blot analysis using the anti-Flag antibody. **c** HA-IKK-β and Flag-TRAF6 were co-transfected with Myc-CHIP into 293 T cells. IP assay was performed using the anti-Flag antibody followed by the Western blot analysis using the HA antibody. The TRAF6/IKK-β interaction was attenuated by CHIP. To prevent TRAF6 degradation, proteasome inhibitor MG-132 was added to the cell culture in the TRAF6/IKK-β co-IP assay.

Our previous study showed that CHIP promotes TRAF6 degradation and inhibits RANKL-induced NF-κB signaling.^12^ To further determine the mechanism by which CHIP regulates TRAF6 signaling, we performed a series of in vitro experiments to determine if CHIP affects TRAF6 interaction with IKK using 293 T cells. We found that CHIP directly interacts with TRAF6 (Fig. 3a). IL-1β enhanced CHIP/TRAF6 interaction (Fig. 3b). Expression of CHIP significantly inhibited TRAF6/IKK-β interaction in 293 T cells (Fig. 3c). These results suggest that CHIP may inhibit TRAF6/IKK-β interaction by inducing TRAF6 degradation.

**Fig. 4:**
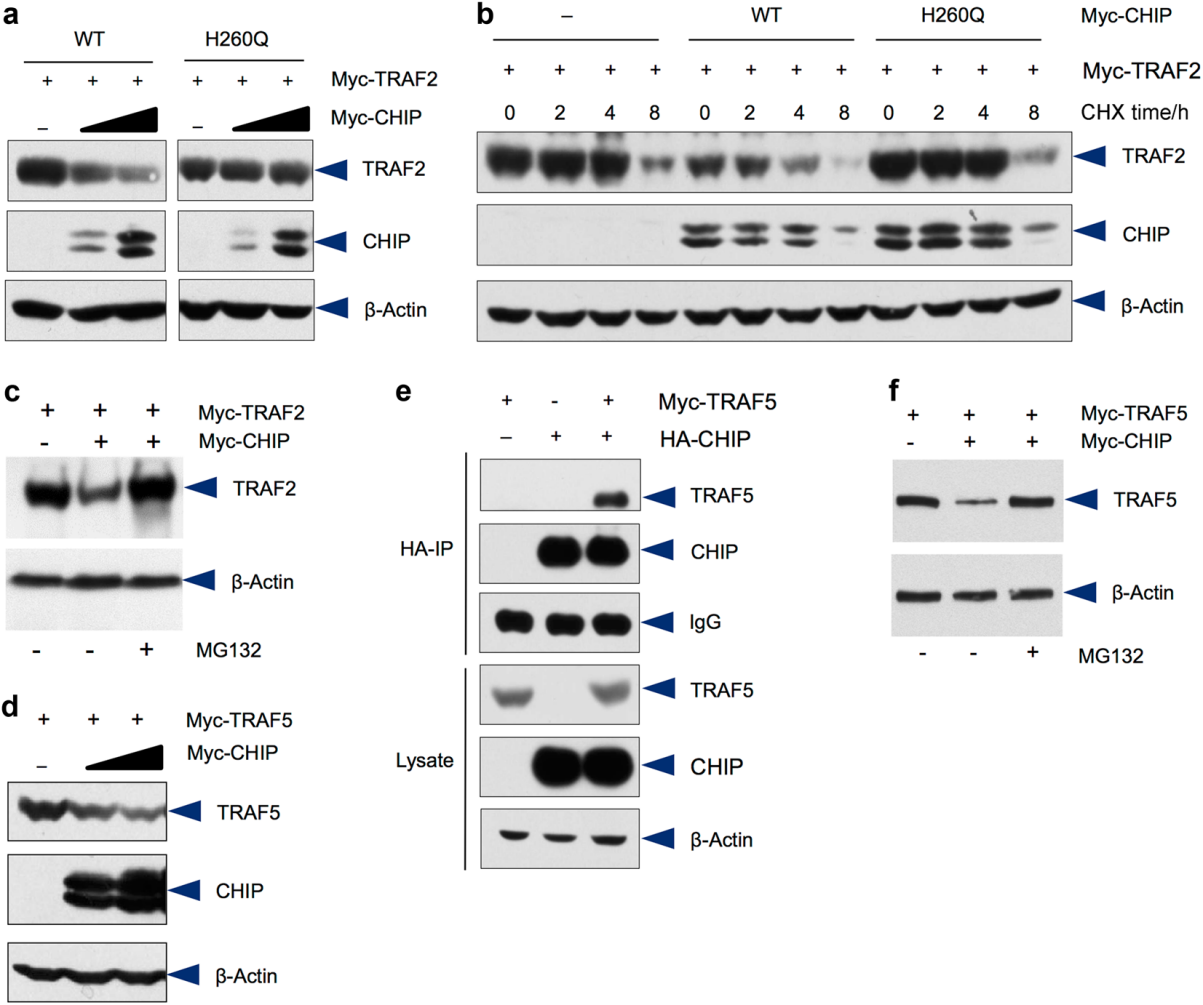
CHIP promotes TRAF2 and TRAF5 degradation. **a** CHIP promotes T RAF2 degradation. Myc-TRAF2 was co-transfected with increasing concentrations of Myc-CHIP or mutant Myc-CHIP (H260Q) in 293 T cells. 24 h after transfection, the cell lysates were collected and samples were subjected to Western blot analysis. TRAF2 protein levels were decreased when cells were transfected with increasing concentrations of CHIP expression plasmid. **b** 293 T cells were transfected with TRAF2 and WT or mutant CHIP expression plasmids and treated with cycloheximide (CHX) for different periods of time as indicated. Transfection of WT CHIP, but not mutant CHIP, accelerated TRAF2 degradation. **c** TRAF2 was transfected with or without CHIP in 293 T cells. Steady state protein levels of TRAF2 were reduced by co-transfection with CHIP. The CHIP-mediated TRAF2 degradation was inhibited by the treatment with proteasome inhibitor MG-132. **d** Myc-TRAF5 and increasing concentrations Myc-CHIP expression plasmids were co-transfected into 293 T cells. CHIP promoted TRAF5 degradation. **e** HA-CHIP and Myc-TRAF5 were co-transfected into 293 T cells. Cell lysates were incubated with the anti-HA antibody and immunoprecipitates were then subjected to Western blot analysis. The results showed that TRAF5 interacts with CHIP. **f** TRAF5 was transfected with or without CHIP in 293 T cells. Steady state protein levels of TRAF5 were reduced by co-transfection with CHIP. The CHIP-mediated TRAF5 degradation was inhibited by the treatment with proteasome inhibitor MG-132.

Given that TRAF2, TRAF3, and TRAF5 belong to the same TRAF family and play critical roles in TNF-α-induced NF-κB signaling, we next examined if CHIP affects stability of TRAF2, TRAF3, and TRAF5. The results of Western blot analysis showed that the protein levels of TRAF2 were decreased when CHIP expression was increased in 293 T cells. In contrast, the mutant CHIP (H260Q), which lacks the E3 ligase ability, failed to affect steady state protein levels of TRAF2 (Fig. 4a). We further examined the half-life of TRAF2 protein by assessing changes in TRAF2 protein levels after treating cells with protein synthesis inhibitor cycloheximide (CHX). The results showed that the half-life of TRAF2 protein was greatly reduced by WT CHIP but not by the mutant CHIP (H260Q) (Fig. 4b). These results indicate that CHIP is a negative regulator of TRAF2 protein. We also examined the proteasome-dependency of CHIP-induced TRAF2 degradation and found that CHIP-mediated TRAF2 degradation could be reversed by the addition of proteasome inhibitor, MG-132 (Fig. 4c), suggesting that CHIP-induced TRAF2 degradation is proteasome-dependent. In addition, protein levels of TRAF5 were also decreased when CHIP expression is increased (Fig. 4d). The IP result demonstrated that TRAF5 was co-precipitated by CHIP in 293 T cells (Fig. 4e). To determine if CHIP-induced TRAF5 degradation is ubiquitin/proteasome-dependent, we treated cells with proteasome inhibitor MG-132. The results showed that CHIP-induced TRAF5 degradation could be reversed by the treatment of MG-132 (Fig. 4f). In contrast, CHIP has no significant effect on TRAF3 degradation (data not shown). Taken together, CHIP regulates protein stability of multiple TRAF family members.

### CHIP inhibits TRAF-mediated NF-κB signaling

**Fig. 5:**
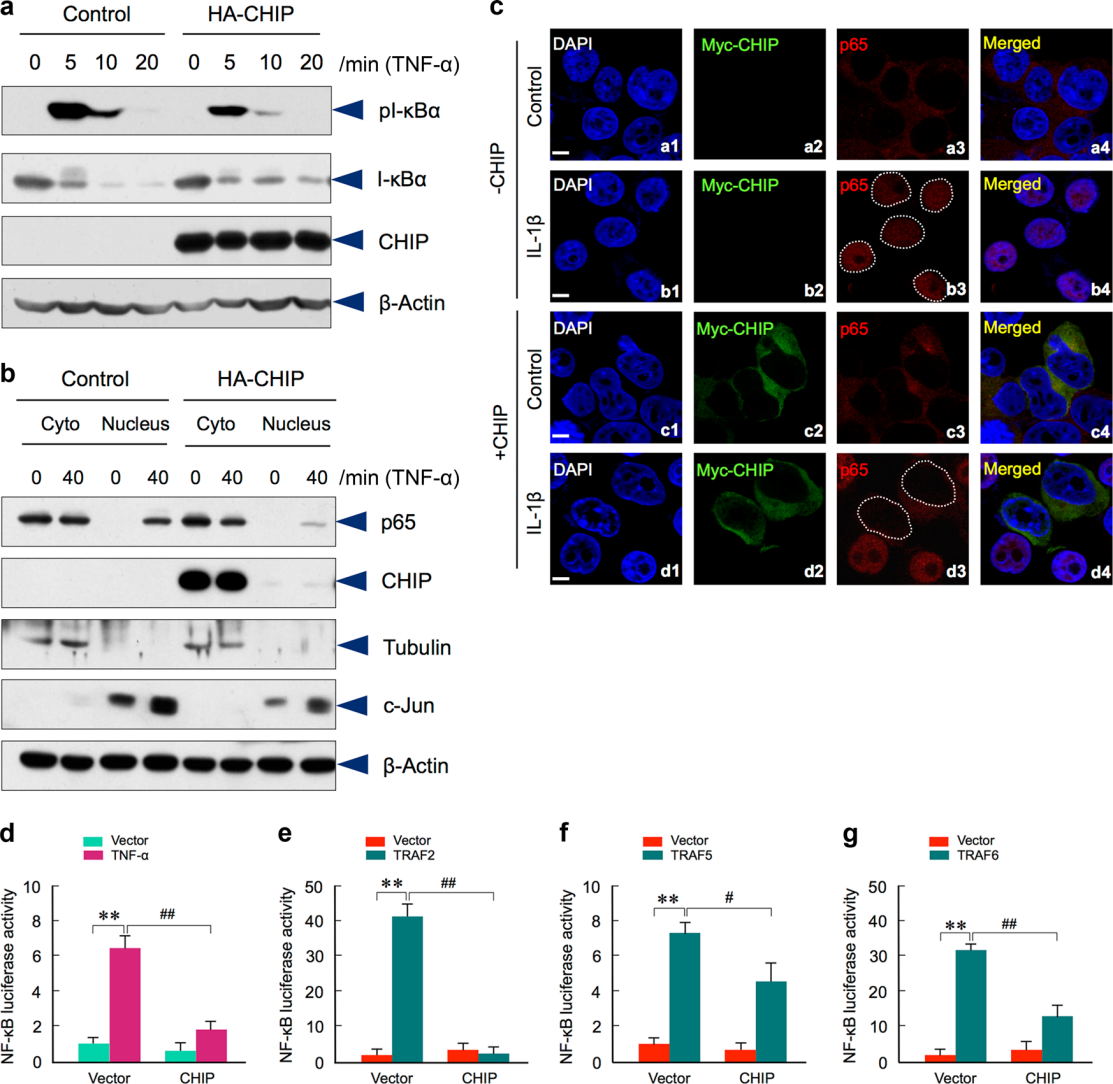
CHIP inhibits TRAF2 and TRAF5-mediated NF-κB signaling. **a** Hela cells stably transfected with HA-CHIP or empty vector were stimulated by TNF-α at indicated time points. The effects of CHIP on endogenous levels of I-κBα and phosphorylated I-κBα were examined. CHIP inhibited TNFα-induced I-κBα phosphorylation. **b** After treatment with TNF-α in Hela cells stably transfected with HA-CHIP or empty vector, p65 protein levels in the cytoplasm (Cyto) and nucleus were detected. CHIP inhibited p65 nuclear translocation. **c** 293 T cells were transfected with or without Myc-CHIP. After treatment with IL-1β for 30 min, the cells were stained with the anti-Myc antibody (green) or anti-p65 antibody (red). The nuclear was stained by DAPI (blue). CHIP inhibited p65 nuclear translocation. The white dotted circles in b3 and d3 showed the nuclear region of cells with or without Myc-CHIP transfection. Scale bar: 10 μm. **d** Luciferase assay was performed in bone marrow stromal (BMS) cells transfected with indicated plasmids and treated with TNF-α for 8 h. CHIP inhibited TNF-α-induced NF-κB signaling. *P < 0.05, **P < 0.01, one-way ANOVA followed by Tukey Post-Hoc test, *n* = 4, compared between control and TNF-α groups; ^##^*P* < 0.01, one-way ANOVA followed by Tukey Post-Hoc test, *n* = 4, compared between empty vector and CHIP transfection groups. **e**–**g** NF-κB reporter construct was transfected into BMS cells with TRAF2, TRAF5 or TRAF6. CHIP inhibited TRAF2, TRAF5 or TRAF6-induced NF-κB signaling. **P < 0.01, one-way ANOVA followed by Tukey Post-Hoc test, *n* = 4, compared between empty vector and TRAF2, TRAF5 or TRAF6 transfected groups; ^#^*P* < 0.05, ^##^*P* < 0.01, one-way ANOVA followed by Tukey Post-Hoc test, *n* = 4, compared between TRAF2, TRAF5 or TRAF6 transfection alone or with CHIP co-transfection group.

Since CHIP regulates TRAF2 and TRAF5 protein levels and TRAF2 and TRAF5 play an important role in TNF-α-induced NF-κB signaling, we then examined whether CHIP affects TNF-α-induced I-κBα phosphorylation and p65 nuclear translocation. The results showed that overexpression of CHIP markedly inhibited the TNF-α-induced I-κBα phosphorylation (Fig. 5a). Notably, the total I-κBα protein levels were increased after CHIP transfection. This is probably because the phosphorylation-induced I-κBα degradation is decreased in CHIP-expressing cells. Next, we performed Western blot analysis using the fractions of the cytoplasm and nuclear proteins and examined changes in p65 localization. The results demonstrated that overexpression of CHIP prevented p65 nuclear translocation when the cells were treated with TNF-α for 40 min (Fig. 5b). These results suggest that CHIP inhibits TNF-α-induced I-κBα phosphorylation. To obtain additional evidence about the role of CHIP in p65 nuclear translocation, we performed immunostaining assay and found that transfection of CHIP inhibited p65 nuclear translocation (Fig. 5c).

To further determine the role of CHIP in NF-κB signaling, we performed NF-κB luciferase assay. The NF-κB reporter construct was transfected with HA-CHIP or empty vectors into BMS cells isolated from 1-month-old WT mice treated with or without TNF-α. The results showed that NF-κB reporter activity was significantly increased by the treatment of TNF-α. However, NF-κB activity was significantly inhibited by the transfection of CHIP (Fig. 5d). To determine the role of CHIP in TRAF2, TRAF5, and TRAF6-regulated NF-κB activity, we transfected TRAF2, TRAF5 or TRAF6 expression plasmids with or without CHIP into BMS cells. The result showed that the TRAF2, TRAF5, and TRAF6-induced NF-κB reporter activity was significantly inhibited by CHIP co-expression (Fig. 5e-g). Taken together, these results suggest that CHIP negatively regulates TRAF2, TRAF5, and TRAF6-induced NF-κB activity.

### CHIP regulates osteoblast gene expression

**Fig. 6:**
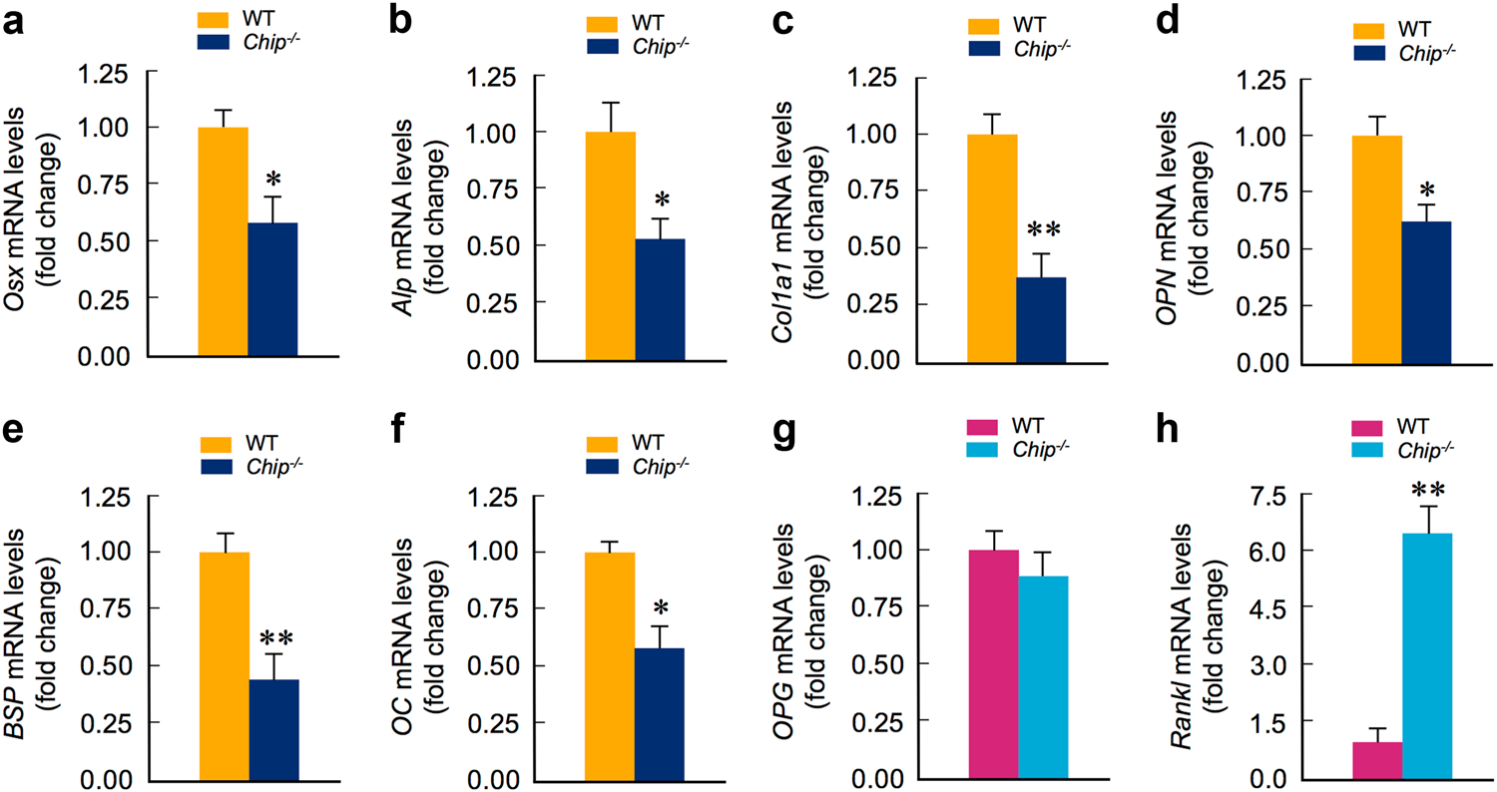
CHIP regulates osteoblast marker gene expression. **a**–**h** mRNA levels of osteoblast and osteoclast marker genes were examined in bone marrow stromal (BMS) cells derived from 1-month-old WT and *Chip*^−/−^ mice. Expression of osteoblast marker genes were significantly decreased in *Chip-*deficient BMS cells. In contrast, *Rankl* expression was significantly increased in *Chip-*deficient BMS cells. *P < 0.05, **P < 0.01, unpaired Student’s *t*-test, *n* = 4.

In an effort to investigate the role of CHIP in osteoblast differentiation, we first examined changes in levels of several proteins critical for osteoblast differentiation, such as β-catenin, Runx2, and Smad3. Previous in vitro studies have shown that CHIP regulates protein stability of these proteins in different cell types.^23,25,31^ Surprisingly, the protein levels of these molecules were not significantly changed in *Chip-*deficient BMS cells (data not shown). We next examined expression of a panel of osteoblast marker genes by the real-time PCR assay. The mRNA levels of *Osterix*(*Osx*) and *alkaline phosphatase (Alp)*were significantly decreased in BMS cells derived from 1-month-old *Chip*^−/−^ mice (Fig. 6a, b). As a consequence, the expression of early osteoblast differentiation markers, such as* type I collagen*(*Col1*) and *osteopontin*(*OPN*), were also significantly decreased in *Chip-*deficient BMS cells (Fig. 6c, d). The expression levels of late stage osteoblast marker genes, such as *bone sialoprotein*(*BSP*) and* osteocalcin*(*OC*), were also significantly reduced in *Chip*-deficient BMS cells (Fig. 6e, f). These data suggest that osteoblast differentiation was impaired in *Chip*^−/−^ mice.

In this study we also examined the mRNA expression of *osteoprotegerin (OPG)* and *Rankl* in BMS cells. We found that *OPG* expression was slightly reduced in *Chip-*deficient BMS cells (Fig. 6g). In contrast, *Rankl* expression was significantly increased in *Chip-*deficient BMS cells (Fig. 6h). These results suggest that reduction in osteoblast differentiation contributes to the bone loss phenotype observed in *Chip*^−/−^ mice.

**Fig. 7:**
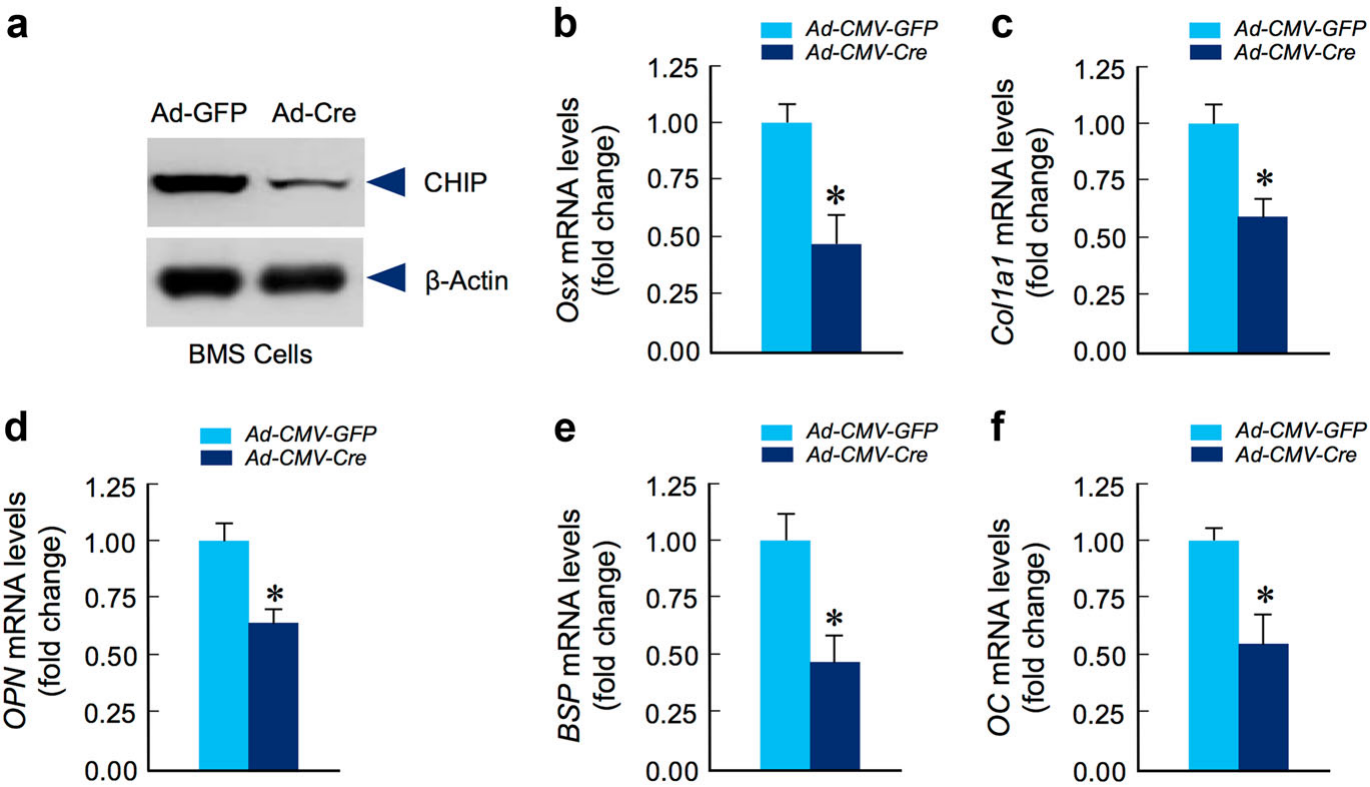
Osteoblast differentiation is inhibited in *Chip*-deficient BMS cells. Bone marrow stromal (BMS) cells were isolated from 1-month-old *Chip*^*flox/flox*^ mice and infected with Ad-CMV-Cre or Ad-CMV-GFP (control virus) and cultured in the presence of osteoblast differentiation medium for 3 days. Expression of CHIP protein was examined by Western blotting (**a**) and expression of osteoblast marker genes was examined by real-time PCR (**b**–**f**). The results showed that expression of osteoblast marker genes, including* osterix*(*Osx*)*,type I collagen*(*Col1a1*)*, osteopontin*(*OPN*)*, bone sialoprotein*(*BSP*) and* osteocalcin*(*OC*) was significantly decreased in *Chip*-deficient BMS cells. *P < 0.05, unpaired Student’s *t*-test, *n* = 4.

To further determine the specific role of CHIP in osteoblast function, we generated *Chip*^*flox/flox*^ mice. We isolated BMS cells from 1-month-old *Chip*^*flox/flox*^ mice and infected these cells with Ad-CMV-Cre virus. The results showed that in vitro deletion of *Chip* in BMS cells significantly decreased CHIP protein levels (Fig. 7a) and inhibited expression of osteoblast marker genes, including *Osx, Col1a1, OPN, BSP,*and *OC*, in *Chip*-deficient BMS cells (Fig. 7b-f).

### CHIP-regulated osteoblast function is NF-κB-dependent

**Fig. 8:**
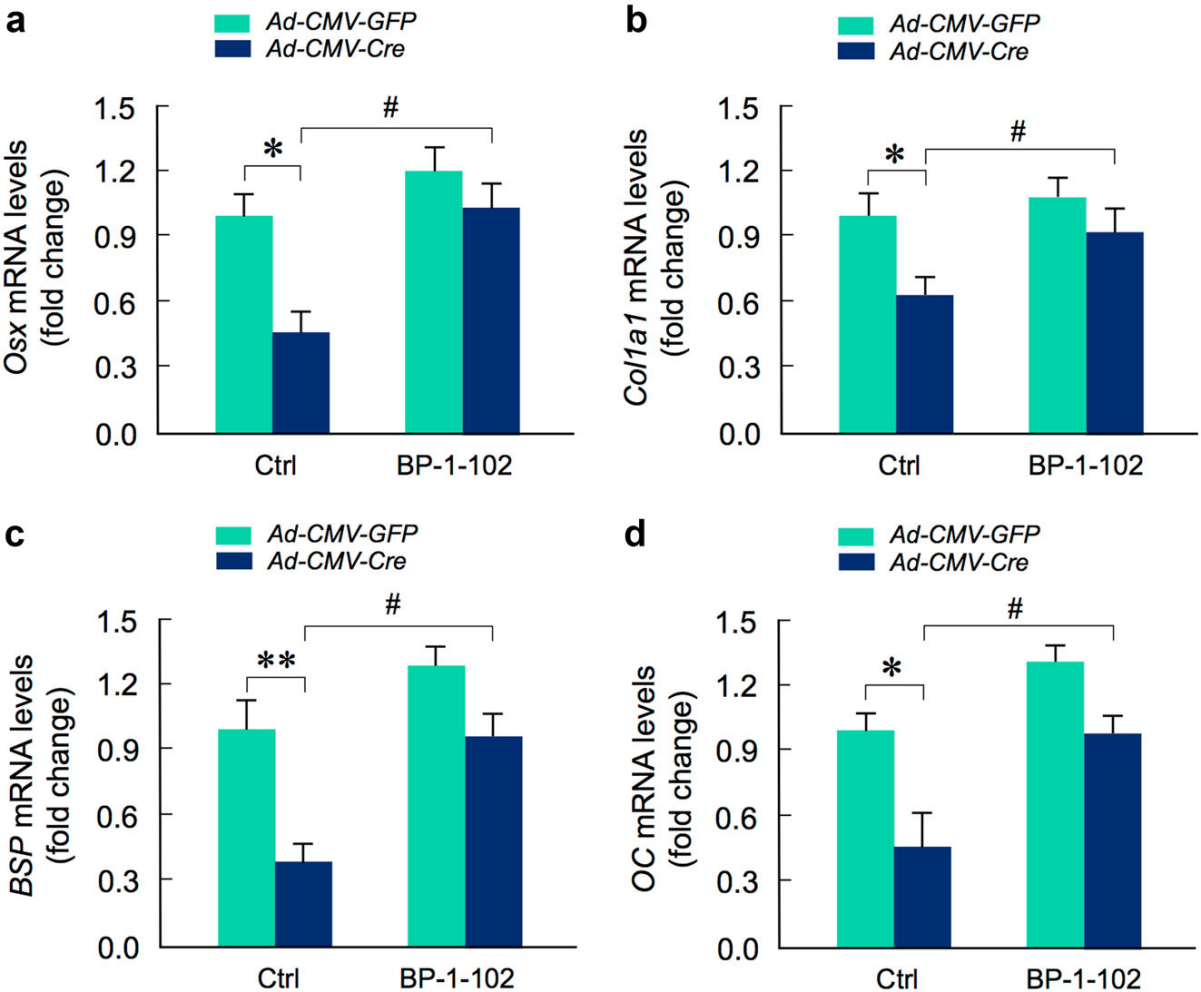
Inhibition of NF-κB signaling reverses the inhibitory effects of osteoblast differentiation observed in *Chip*-deficient BMS cells. Bone marrow stromal (BMS) cells were isolated from 1-month-old *Chip*^*flox/flox*^ mice and infected with Ad-CMV-Cre or Ad-CMV-GFP (control virus) and cultured with osteoblast differentiation medium for 3 days in the presence or absence of NF-κB inhibitor, BP-1-102. Expression of osteoblast marker genes, including *Osx, Col1a1, BSP,*and* OC* was inhibited in *Chip*-deficient BMS cells. Addition of NF-κB inhibitor (BP-1-102) reversed inhibitory effects of expression of osteoblast marker genes observed in *Chip*-deficient BMS cells. *P < 0.05, **P < 0.01, one-way ANOVA followed by Tukey Post-Hoc test, *n* = 4, compared between WT and *Chip*-deficient BMS cells. ^#^*P* < 0.05, one-way ANOVA followed by Tukey Post-Hoc test, *n* = 4, compared between *Chip*-deficient BMS cells treated with or without BP-1-102.

To determine if *Chip*-deficiency mediated inhibition of osteoblast differentiation is NF-κB-dependent, we isolated BMS cells from 1-month-old *Chip*^*flox/flox*^ mice and infected these cells with Ad-CMV-Cre or Ad-CMV-GFP (control virus) and treated cells with or without NF-κB inhibitor, BP-1-102. We examined changes in expression of several osteoblast marker genes in these cells and found that treatment with NF-κB inhibitor significantly reversed the inhibitory effects on the expression of osteoblast marker genes observed in *Chip*-deficient BMS cells (Fig. 8). BP-1-102 inhibits nuclear NF-κB retention both in vitro and in vivo, thereby attenuating NF-κB activation.^32^ These results suggest that CHIP regulates osteoblast differentiation through a NF-κB-dependent mechanism.

## Discussion

Bone remodeling is a highly coordinated process involving bone resorption and bone formation. The dynamic process of bone remodeling was impaired in *Chip*^−/−^ mice, suggesting that CHIP plays a critical role in bone remodeling. Our previous findings showed that the bone loss phenotype observed in *Chip*^−/−^ mice is due to abnormally increased osteoclast formation and bone resorption. In the present studies we provided novel evidence that in addition to the enhanced osteoclast formation, *Chip* deficiency in mice also leads to reduced bone formation.

NF-κB and its signaling molecules play critical roles in regulation of osteoclast formation^13–16^ and osteoblast function,^17–22^ and TRAF family members are critical mediators in NF-κB signaling.^7–10^ Our previous study showed that TRAF6 protein levels are increased in *Chip-*deficient osteoclasts and we have characterized the effect of CHIP on regulation of TRAF6 protein stability.^12^ In the present studies, we further investigated the role of CHIP in TRAF6/IKKβ interaction and TRAF2 and TRAF5 protein stability and functions. We found that 1) CHIP interferes with TRAF6/IKKβ interaction; 2) CHIP induces TRAF2 and TRAF5 protein degradation; 3) CHIP inhibits TRAF2 and TRAF5-induced NF-κB signaling. These results suggest that CHIP functions as a critical regulator in NF-κB signaling through regulation of multiple TRAF family members.

Osteoblast/osteoclast cross talk regulates bone formation and bone resorption with remarkable precision. For example, the key regulator for osteoclast bone resorption is RANKL, which expressed by activated osteoblasts.^33^ Its action on the RANK receptor in osteoclasts is regulated by OPG, a decoy receptor, which is also expressed in osteoblasts.^34^ In *Chip-*deficient BMS cells, *Rankl* expression was significantly increased (Fig. 6j). These results demonstrate that in addition to the direct regulation of bone resorption via promoting TRAF degradation, CHIP also indirectly enhances osteoclast formation via controlling osteoblast/osteoclast cross talk.

Although previous studies demonstrated that CHIP regulates protein stability of β-catenin, Runx2, and Smad3 in vitro,^23,25,31^ in the present studies, we did not detect significant changes in the steady state protein levels of these molecules, except slight reduction of β-catenin protein levels in *Chip-*deficient BMS cells (data not shown). It is known that activation of NF-κB signaling inhibits osteoblast function;^17–22^ however, the detail mechanism remains undefined. Recent studies showed that transcriptional enhancers of cell type specific genes could serve as signal integration hubs binding to signal-dependent transcription factors, such as NF-κB, and lineage-determining transcription factors, such as Runx2, to control the gene expression program and determine cell differentiation fate.^35,36^ This may explain why NF-κB regulates osteoblast function without altering Runx2 protein levels.

It is well known that NF-κB signaling plays an important role in inflammation and in rheumatoid arthritis (RA).^37,38^ In addition to the inflammation, activation of NF-κB signaling also leads to bone loss by upregulation of osteoclast formation and inhibition of osteoblast function. In the present studies, we demonstrated that CHIP inhibits NF-κB signaling by inducing the degradation of multiple TRAF family members, including TRAFs 2, 5, and 6. In *Chip*^−/−^ mice osteoclast formation was increased^12^ and osteoblast differentiation was inhibited, demonstrated by the present studies. These findings suggest that CHIP may play an important role in the development of RA disease. Expression or function of CHIP may be inhibited during RA development, leading to activation of NF-κB signaling and bone loss phenotype in joint tissues due to increased osteoclast formation and reduced osteoblast function. To further determine the specific role of CHIP in joint tissues, we have recently generated *Chip*^*flox/flox*^ mice. Using tissue-specific knockout approach, we will further dissect the specific effects of CHIP in specific cell populations in bone and cartilage tissues, including mesenchymal stem cells (MSCs), osteoblast and osteoclast lineage cells, and in synovial cells.

In summary, in this study we demonstrate that bone loss phenotype observed in *Chip*^−/−^ mice is due to the combination of increased bone resorption and reduced bone formation. Since abnormal increase in osteoclast formation and reduced osteoblast function may lead to bone and joint diseases, our findings suggest that CHIP may serve as a novel target for the drug development to treat bone loss associated diseases.

## Materials and methods

### Mice

*Chip* knockout (KO) mice were obtained from NIH. The first three coding exons of the *Chip* gene were targeted by homologous recombination. Both wild-type (WT) and *Chip*^−/−^ mice were maintained in a C57BL/6 and 129SvEv background. *Chip*^*flox/flox*^ mice were generated in Nanjing Biomedical Research Institute of Nanjing University, Nanjing, China. In these mice the *Chip* gene was floxed at the flanking sites of exon 1 and exon 3.

### Quantitative real-time PCR

Total RNA was extracted from BMS cells which were isolated from 1-month-old *Chip*^−/−^ mice and WT littermates or BMS cells derived from *Chip*^*flox/flox*^ mice infected with Ad-CMV-Cre or Ad-CMV-GFP (control virus). cDNA was synthesized from 1 μg of RNA using the iScript cDNA synthesis kit (Bio-Rad). Real-time PCR analysis was performed using primers for detecting osteoblast and osteoclast marker genes, including *osterix* (*Osx), type I collagen*(*Col1*)*,alkaline phosphatase*(*Alp*)*, OPN,osteocalcin*(*OC*)*, osteoprotegerin*(*Opg*)*, receptor activator of NF-κB ligand* (*Rankl*), and *Ki67*.

### Luciferase assays

BMS cells were transiently transfected with pGL3/NF-κB-luc reporter and expression plasmids as indicated. The luciferase assay was performed to examine the effect of CHIP in NF-κB signaling.

### Histology

Tibiae and vertebrae were harvested and fixed in 10% NB-formalin for 3 days and decalcified for 14 days in 14% EDTA and then paraffin-embedded. Three micrometers sections were cut and Alcian blue/H&E Orange G and Safranin O/Fast green staining and TRAP staining were performed.

### Plasmids and reagents

pCMV/Myc-TRAF6, TRAF2, and TRAF5 was provided by Dr. Lingqiang Zhang (State Key Laboratory of Proteomics, Beijing, China). pEFNeo/HA-CHIP, pRKIM/Myc-CHIP, and pRKIM/Myc-CHIP (H260Q) were generated in our lab as described previously.^23^ I-κBα, phosphor-I-κBα (Ser32) and anti-α-tubulin (3873p) were purchased from Cell Signaling Technology (Beverly, MA). Anti-TRAF6 (H-274) and anti-p65 (C-20) antibodies were purchased from Santa Cruz Biotechnology (Santa Cruz, CA). Anti-CHIP antibody was developed in our lab.^16^ MG132 was purchased from Millipore (Billerica, MA). Cyclohexanone (CHX) was purchased from Amresco (AMRESCO Inc., OH).

### Cell culture

293 T cells and Hela cells were cultured in DMEM supplemented with 10% fetal calf serum (FCS) at 37 °C under 5% CO_2_. In CHIP/TRAF5 co-IP assay, proteasome inhibitor MG-132 was added (10 μM). BMS cells were isolated from 1-month-old *Chip*^*flox/flox*^ mice and cultured with αMEM and 10% FCS. BMS cells were then infected with Ad-CMV-Cre or Ad-CMV-GFP (control virus) in the presence of osteoblast differentiation medium for 3 days.^39^ Expression of osteoblast marker genes was examined by real-time PCR assay. For mineralized bone matrix formation assay, BMS cells were cultured with osteoblast differentiation medium with BMP-2 (50 ng·mL^-1^) for 2 weeks. Alizerin red staining was then performed. NF-κB inhibitor, BP-1-102, was obtained from Calbiochem (EMD Millipore, Billerica, MA).

### Immunoprecipitation and Western blot analysis

The immunoprecipitation (IP) and Western blot assays were performed as previously described.^12,23^

### Alizarin red staining

BMS cells were isolated from femur and tibia of 1-month-old WT and *Chip*^−/−^ mice (or *Chip*^*flox/flox*^ mice infected with Ad-CMV-Cre or Ad-CMV-GFP), and were cultured with αMEM supplemented with 10% FCS and treated with ascorbic acid (50 μg·mL^-1^) and β-glycerophosphate (10 mmol·L^-1^) and BMP-2 (50 ng·mL^-1^) for 2 weeks. At the end of the cell culture, BMS cells were fixed in 10% formalin and Alizarin red staining was performed.^40,41^

### Calcein labeling

Calcein (20 mg·kg^-1^, i.p.) labeling was performed 1 and 4 days before 1-month-old WT and *Chip*^−/−^ mice were sacrificed. At the end of the experiments, tibiae were removed, fixed in 75% ethanol, and embedded in plastic. Transverse sections were cut at 5-μm thickness and unstained sections were viewed under fluorescent microscope.^40,42^
